# Response: Commentary: Preoperative status of gut microbiota predicts postoperative delirium in patients with gastric cancer

**DOI:** 10.3389/fpsyt.2022.991290

**Published:** 2022-09-09

**Authors:** Hu Liu, Xue-sheng Liu

**Affiliations:** Department of Anesthesiology, Key Laboratory of Anesthesiology and Perioperative Medicine of Anhui Higher Education Institutes, First Affiliated Hospital of Anhui Medical University, Anhui Medical University, Hefei, China

**Keywords:** gut microbiota, postoperative delirium, 16S rRNA, principle coordinate analysis, gastric cancer

## Introduction

We appreciate to receive an insightful comment by Tan et al. Our study preliminarily investigated the association between post-operative delirium (POD) and gut microbiota. We observed multiple differentially abundant bacteria between the patients with and without POD. The results indicated that there were significant associations between the pathogenesis of POD and composition of the gut microbiota. More details about bacterial types can be found in the context.

We admitted that the sample size was relatively small, so we noted the limitation in the discussion (1). Theoretically, the sample size should be set to meet the power value of statistics. We totally collected almost 100 samples during the study, but some samples were excluded from the final experiment of 16S rRNA sequencing because of restrictive conditions. These conditions helped the elimination of bias, such as surgical type, antibiotics application within the past 6 months before sampling, etc. Meanwhile, some samples were also excluded from quality control during the experiment. So the final sample size for experiment and analysis was 20 POD patients and 20 non-POD patients.

Tan et al. proposed another suggestive comment for the difference of delirium rate between anesthetic types. Previous studies have shown controversial results on the delirium rate between propofol anesthesia and sevoflurane anesthesia. For example, Isshii et al. (2) reported that the incidence of POD in sevoflurane anesthesia was significantly higher than that in propofol anesthesia; However, Mei et al. (3) and Nishikawa et al. (4) found no statistical differences in POD between the two anesthetics. It could reduce the bias to control the single variable for anesthetic. Therefore, we intend to perform a stratification analysis on POD with an augmented sample size. Second, in the β-diversity analysis, we performed Principal Component Analysis (PCA) and Principal Co-ordinates Analysis (PCoA) analyses to investigate the comparability of samples from the two cohorts. The results indicated that the *P*-values for PCA and PCoA were both >0.05. Given that fecal samples were derived from patients with gastric cancer, these results indicated comparability between the two cohorts. The analyses in our study were just performed according to an established analytical procedure (2). The quotiety of explanations on the X-axis in PCA and PCoA were missed, so we added the percentages in this letter: PCA1 (23%) in PCA analysis and PCoA1 (22.49%) in PCoA analysis.

Furthermore, we apologize for the mistake of the registration number, which should be changed from ChiCTR200030131 to ChiCTR2000030131. This study belongs to a sub-project of the clinical interventional trial.

Our study presented a preliminary investigation on the potential functions of “brain-gut” axis on POD. We are grateful for the reader's concern about the novelties and the limitations. We would like to make further studies on both clinical association and mechanism, and wish to develop a multi-center collaboration.

## Author contributions

HL wrote the draft and X-sL rewrote it. All authors read and approved the final manuscript.

## Conflict of interest

The authors declare that the research was conducted in the absence of any commercial or financial relationships that could be construed as a potential conflict of interest.

## Publisher's note

All claims expressed in this article are solely those of the authors and do not necessarily represent those of their affiliated organizations, or those of the publisher, the editors and the reviewers. Any product that may be evaluated in this article, or claim that may be made by its manufacturer, is not guaranteed or endorsed by the publisher.
